# Genomic HIV RNA Induces Innate Immune Responses through RIG-I-Dependent Sensing of Secondary-Structured RNA

**DOI:** 10.1371/journal.pone.0029291

**Published:** 2012-01-03

**Authors:** Randi K. Berg, Jesper Melchjorsen, Johanna Rintahaka, Elisabeth Diget, Stine Søby, Kristy A. Horan, Robert J. Gorelick, Sampsa Matikainen, Carsten S. Larsen, Lars Ostergaard, Søren R. Paludan, Trine H. Mogensen

**Affiliations:** 1 Department of Infectious Diseases, Aarhus University Hospital - Skejby, Aarhus, Denmark; 2 Unit of Excellence for Immunotoxicology, Finnish Institute of Occupational Health, Helsinki, Finland; 3 Department of Biomedicine, University of Aarhus, Aarhus, Denmark; 4 AIDS and Cancer Virus Program, SAIC-Frederick, Inc., National Cancer Institute-Frederick, Frederick, Maryland, United States of America; Institut Pasteur Korea, Republic of Korea

## Abstract

**Background:**

Innate immune responses have recently been appreciated to play an important role in the pathogenesis of HIV infection. Whereas inadequate innate immune sensing of HIV during acute infection may contribute to failure to control and eradicate infection, persistent inflammatory responses later during infection contribute in driving chronic immune activation and development of immunodeficiency. However, knowledge on specific HIV PAMPs and cellular PRRs responsible for inducing innate immune responses remains sparse.

**Methods/Principal Findings:**

Here we demonstrate a major role for RIG-I and the adaptor protein MAVS in induction of innate immune responses to HIV genomic RNA. We found that secondary structured HIV-derived RNAs induced a response similar to genomic RNA. In primary human peripheral blood mononuclear cells and primary human macrophages, HIV RNA induced expression of IFN-stimulated genes, whereas only low levels of type I IFN and tumor necrosis factor α were produced. Furthermore, secondary structured HIV-derived RNA activated pathways to NF-κB, MAP kinases, and IRF3 and co-localized with peroxisomes, suggesting a role for this organelle in RIG-I-mediated innate immune sensing of HIV RNA.

**Conclusions/Significance:**

These results establish RIG-I as an innate immune sensor of cytosolic HIV genomic RNA with secondary structure, thereby expanding current knowledge on HIV molecules capable of stimulating the innate immune system.

## Introduction

HIV is a retrovirus that targets mononuclear cells of the immune system and establish lifelong infection with progressive immunodeficiency, susceptibility to opportunistic infections, and the development of AIDS if left untreated [1]. The natural history of HIV infection is characterized by an acute infection with high levels of viraemia and irreversible damage to the immune system, in particular the gut associated lymphoid tissue. This is followed by a chronic phase with persistent immune activation and depletion of CD4 T cells, ultimately resulting in progressive immune exhaustion and profound immunodeficiency [2]–[5]. Based on the observation that initiation of highly active antiretroviral therapy leads to a rapid decline in immune activation, which is correlated with a significant reduction in HIV viraemia, a direct contribution of HIV particles to immune activation has been proposed [6], [7].

Since one of the fundamental characteristics of HIV pathogenesis is the failure of the immune system to initially recognize and control viral infection, early events taking place during the very first hours and days of infection are likely to be of central importance. The innate immune system constitutes the first line of defence against invading pathogens and is also a prerequisite for the subsequent activation and maturation of adaptive immune responses [8], [9]. PRRs have been assigned a central role in innate immune responses due to their ability to recognize and respond to evolutionarily conserved structures on pathogens termed PAMPs [10]. Activation of PRRs induces a proinflammatory and antimicrobial response by triggering signal transduction pathways involving NF-κB and the MAPK pathway, as well as IFN regulatory factors (IRF)s ultimately resulting in the synthesis of cytokines, chemokines, and antiviral type I IFN [11], [12].

PRRs are divided into several families, including TLRs, retinoid acid inducible gene (RIG)-like receptors (RLR)s, nucleotide-binding and oligomerization domain-like receptors, and most recently emerging families of DNA receptors [11], [12]. In the context of viral infection, the receptors for nucleic acids are particularly important [13]. Among the TLRs, the endosomally located TLRs sense foreign nucleic acids with TLR3 recognizing dsRNA and TLR7/8 sensing ssRNA, whereas TLR9 is activated by unmethylated DNA [11], [14]–[17]. In the cytosolic compartment, the RLRs RIG-I and melanoma-differentiation-associated gene 5 are RNA helicases that play a pivotal role in sensing of cytoplasmic RNA [18]. Studies have suggested differential roles of these receptors with RIG-I being responsible for recognizing 5′triphosphorylated RNA and short dsRNAs (e.g. stem-loop structures [19]–[21], whereas melanoma-differentiation-associated gene 5 responds to long dsRNA and higher order RNA structures [21], [22]. Finally, cytosolic DNA receptors, which sense most types of dsDNA, have been identified [23]–[27].

During the replication cycle of HIV, the HIV genome consisting of two identical copies of positive strand ssRNA together with the viral capsid is introduced into the cytoplasm of the cell. During the process of reverse transcription, RNA∶DNA hybrids are generated, followed by cDNA and finally dsDNA, which is transported into the nucleus and integrated into the host cell DNA genome. Later during the HIV replication cycle, viral genomic ssRNA and mRNA, the latter of which encodes structural proteins, are synthesized and present in the cytoplasm [28]. Thus, several of the above mentioned PRRs may theoretically be involved in recognizing various HIV nucleic acid structures and trigger innate immune responses. The fact that HIV replication takes place in the cytosolic compartment allows for HIV PAMPs either present in the viral genome or synthesized during the viral replication cycle to be be recognized by cytosolic PRRs.

One of the first links between HIV and PRRs was obtained from a study demonstrating that U-rich ssRNA derived from HIV is recognized by TLR7/8 and stimulates DCs and macrophages to secrete IFN-α and proinflammatory cytokines [16]. In agreement with this, MyD88-dependent activation of plasmacytoid DCs (pDC)s and monocytes by U-rich ssRNA from the HIV long terminal repeat (ssRNA40) has been demonstrated [29]. The demonstration that endocytosis of HIV particles and the presence of viral nucleic acids in the endocytic compartment is required for pDC activation and IFN-α secretion further supports a role for endosomal TLRs, including TLRs 3, 7/8, and 9 [30]. Additionally, histopathological studies in mice have revealed that sustained TLR7/8-triggering results in lymphopenia, abolished antibody production, and alterations in lymphoid micro-architecture resembling HIV-mediated pathology [31]. TLR7 has also been attributed an important role in a recent study by Lepelley *et al.*, in which evidence was presented suggesting innate sensing of HIV-infected lymphocytes by both endosomal TLR7-mediated- and cytoplasmic pathways, the latter of which was dependent on incoming viral material and IRF3 [32]. Although the specific cytosolic viral sensor was not identified, the work adds to a number of other reports providing strong support for PRRs other than TLRs in HIV recognition [32], [33]. Recently, Hiscott and colleages demonstrated that purified genomic RNA from HIV is detected by the cytosolic RNA-sensor RIG-I and induces a type I IFN response [34]. However, evasion strategies in human monocyte-derived macrophages seem to evade such responses during HIV infection [34]. Regarding sensing of HIV DNA, Stetson *et al.* have demonstrated that retroviral cDNA activates a type I IFN response through an unidentified DNA receptor [35]. More recently, Lieberman and associates presented evidence that the cytosolic exonuclease TREX1 inhibits the innate immune response to HIV by degrading ssDNA derived from integrated provirus [36]. It was demonstrated that in the absence of TREX1, a type I IFN response is induced that inhibits HIV replication and spreading, although the specific DNA sensor responsible remains to be identified [36]. Finally, a cell-intrinsic sensor for HIV has been identified which mediates an antiviral immune response in DCs dependent on the interaction with newly synthesized HIV capsid and cellular cyclophilin A [37].

Here, we have investigated the innate immune response induced by HIV genomic RNA in PBMCs. We report that HIV genomic RNA and HIV-derived secondary structured RNA localize to peroxisomes to induce innate immune responses with low induction of type I IFN and higher induction of IFN-stimulated genes (ISG)s by a mechanism dependent on RIG-I and its down-stream adaptor protein MAVS.

## Results

### Genomic HIV RNA induces innate immune responses in PBMCs

**Figure 1:**
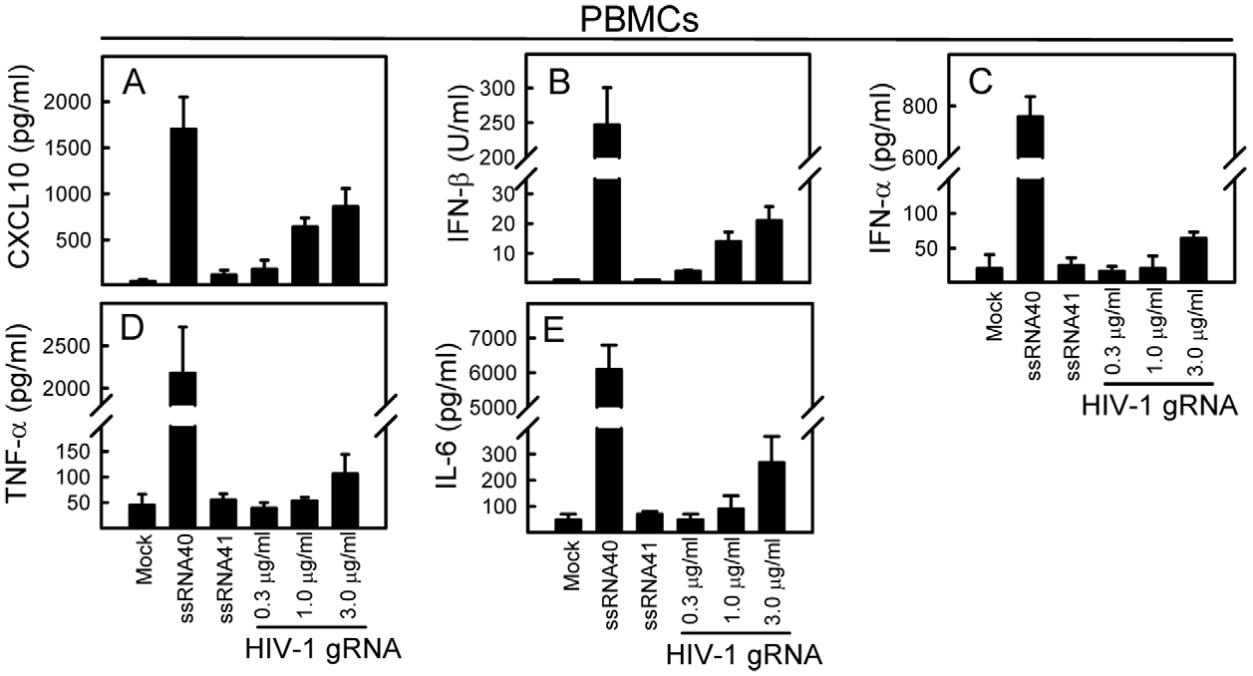
Genomic HIV RNA induces innate immune responses dominated by ISGs. PBMCs were stimulated for 16 h with HIV genomic RNA in increasing doses (from 0.3 to 3.0 µg/ml). Positive and negative controls included ssRNA40 (2 µg/ml) and ssRNA41 (2 µg/ml), respectively. Supernatants were harvested for measurement of (A) CXCL10, (B) IFN-β, (C) IFN-α, (D) TNF-α, and (E) IL-6. Data are shown as means of triplicates +/− st.dev. Similar results were obtained in two or three independent experiments. Mock, Lipofectamine 2000 alone.

Previous studies have identified a U-rich ssRNA sequence derived from the HIV long terminal repeat (ssRNA40) with a capacity to activate pDCs and monocytes through TLR7 and 8 [16], [29]. However, the immuno-stimulatory potential of the entire HIV genome has never been assessed in primary human cells. We therefore transfected purified virion-derived HIV genomic RNA into human PBMCs. As shown in Fig. 1A, HIV genomic RNA induced expression of the chemokine CXCL10 in a dose-dependent manner in PBMCs. Although the induction of CXCL10 was weaker than the one observed using ssRNA40, which is on a thio-ester backbone, we observed a robust induction, when the cells we treated with 3 µg/ml of RNA. In addition, introduction of RNA isolated from HIV virions into the cytoplasm of cells led to modest induction of IFN-β (Fig. 1B). Finally, HIV genomic RNA affected IFN-α, TNF-α, and IL-6 levels to only marginal extent (Fig. 1 C–E).

### RNA oligos derived from the HIV genome induce innate immune responses in PBMCs

**Figure 2:**
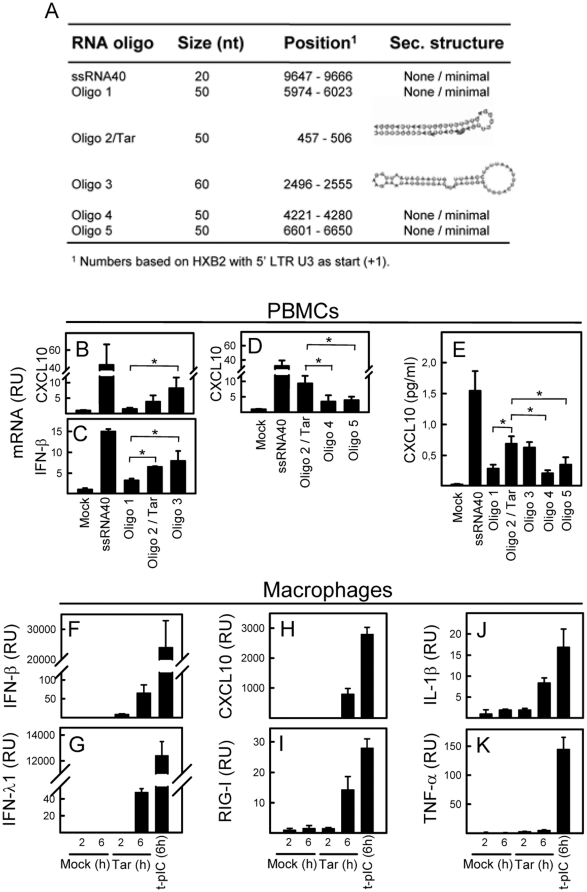
HIV RNA oligos derived from the HIV genome induce an innate immune response in human PBMCs and primary macrophages. (A) Schematic illustration of the size, position and secondary structure of the HIV-derived RNA oligos used in this study. (B-E) PBMCs were stimulated with different RNA oligos derived from the HIV genome (2 µg/ml) or with ssRNA40 (2 µg/ml). Total RNA and supernatants were harvested after 6 and 20 h, respectively, and CXCL10 (mRNA and protein) and IFN-β (mRNA) levels were measured. Data are shown as means of triplicates +/− st.dev. Similar results were obtained in three independent experiments. (F–K) Primary macrophages were differentiated from human PBMCs and stimulated for either 2 or 6 h with HIV Tar (oligo 2, 2 µg/ml). Transfected poly(I:C) (t-pIC) was included as a positive control (2 µg/ml). Total RNA was isolated at the indicated time points and analyzed by qPCR for the presence of IFN-β (E), IFN-λ1 (F), CXCL10 (G), RIG-I (H), IL-1β (I), and TNF-α (J). Data are presented as mean values from analysis of 2 independent donors +/− st.dev. Mock, Lipofectamine 2000 alone. RU, relative units. *, p<0.05.

A structural model of the HIV genome has recently been reported, suggesting a high degree of secondary structure [38]. To examine if the secondary structured regions of the HIV RNA genome may contribute to its immuno-stimulatory activity, we designed HIV-derived RNA oligos with varying degrees of secondary structure based on biophysical predictions in RNAfold (Fig. 2A). When comparing the ability of these RNA oligos to induce CXCL10 and IFN-β expression in PBMCs we found that the RNA oligos with secondary structure (Oligo 2/Tar and oligo 3) induced significantly higher amounts of CXCL10 and IFN-β mRNA than oligo 1, 4, and 5, which were predicted not to possess extensive secondary structure (Fig. 2B–D). The same observation was done when CXCL10 expression was examined at the protein level (Fig. 2E).

In order to characterize the innate response induced by secondary structured HIV RNA, we performed stimulation of primary human monocyte-derived macrophages with Tar. By RT-qPCR analysis we found that Tar potently induced expression of the ISGs CXCL10 and RIG-I within 6 hours of stimulation (Fig. 2H–I), and also stimulated expression of IL-1β mRNA to largely the same extent as the dsRNA mimic Polyinosinic∶polycytidylic acid (poly(I∶C)), which was used as a positive control (Fig. 2J). Although detectable, more modest levels of type I (IFN-β) and type III IFN (IFN-λ1) were induced by HIV Tar compared to Poly(I∶C) (Fig. 2F–G), and the levels of TNF-α mRNA were only affected marginally by the HIV-derived RNA (Fig. 2K).

### HIV RNA activates the NF-κB, p38, and IRF signaling pathways

**Figure 3:**
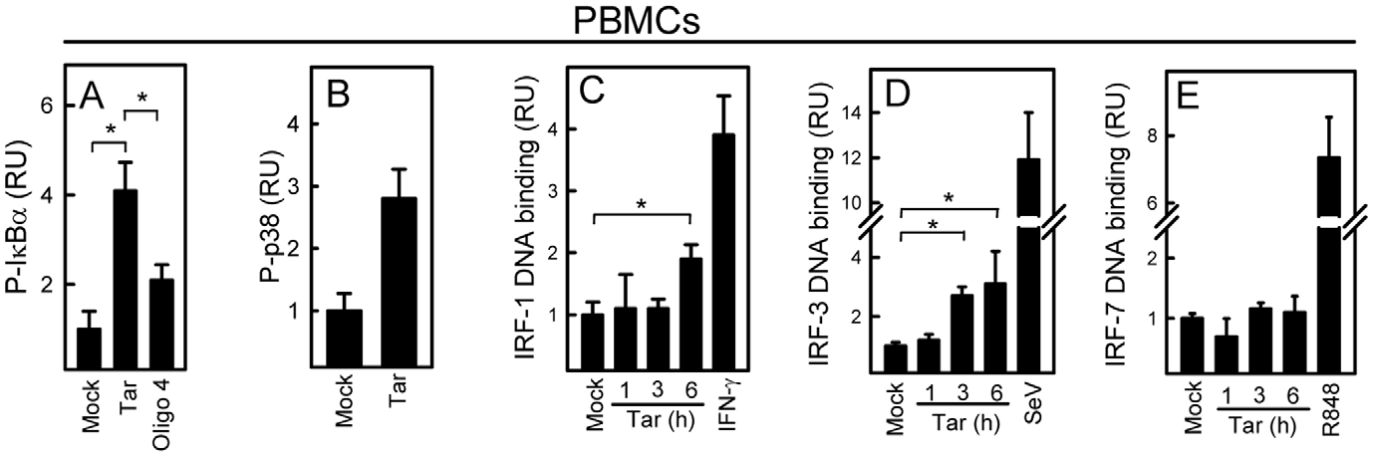
HIV RNA activates the NF-κB, p38, and IRF signaling pathways. PBMCs were stimulated with HIV Tar (oligo 2, 2 µg/ml) and in panel A also with oligo 4 (2 µg/ml). (A–B) Whole-cell lysates were isolated 2 h post treatment, and (C–E) nuclear extracts were isolated at the indicated time points (IFN-γ, 10 ng/ml, 6 h; SeV, MOI 1, 6 h; R848, 500 ng/ml, 6 h). (A–B) P-IκBα and P-p38 were measured by Luminex technology and (C–E) DNA binding of IRF-1, 3, and 7 to an ISRE consensus sequence was measured by TransAM. Data are shown as means of duplicates or triplicates +/− st.dev. Similar results were obtained in two or three independent experiments. RU, relative units. Mock, Lipofectamine 2000 alone. *, p<0.05.

To characterize signaling triggered by HIV-derived RNA, we next transfected human PBMCs with HIV Tar and oligo 4 and harvested whole-cell extracts. As shown in Fig. 3A and B, Tar induced phosphorylation of IκBα and p38 corresponding to activation of the NF-κB and MAPK pathways, respectively. Importantly, Tar RNA activated the NF-κB pathway to a significantly higher extent than oligo 4 did (Fig. 3A).

With the apparent preference for induction of ISGs by HIV RNA, we were particularly interested in examing the ability of HIV Tar to activate transcription factors of the IRF family. Nuclear extracts were isolated from cells treated for 0, 1, 3, and 6 h and the levels of binding of IRF-1, 3, and 7 to the ISRE consensus sequence was determined by TransAM. As shown in Fig. 3 C–E, stimulation with HIV Tar led to a modest activation of DNA binding by IRF-1, a more robust activation of IRF-3, and no detectable activation of IRF-7.

### HIV Tar localizes to peroxisomes but not to mitochondria

**Figure 4:**
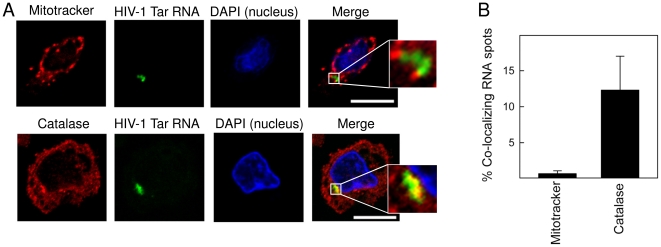
HIV Tar co-localizes with peroxisomes but not with mitochondria. PBMCs stimulated with 1.2 µg/ml FAM-labeled HIV-Tar RNA (green) for 180 min were labeled for mitochondria (red) or peroxisomes (red). Nuclei were stained with DAPI (blue). Co-localization is shown in yellow, scale bar 20 µm. Representative images are shown in A, while panel B shows a quantification of percentage RNA-organelle co-localization as obtained by counting more than 100 cells per group. Similar results were obtained in two independent experiments.

Several publications within the last few years have underscored the importance of the cytosolic context and involvement of different organelles in the final outcome of cellular stimulation with different PAMPs [39]–[41]. Most recently, signaling mediated by RLRs from peroxisomes has been reported to result in an innate response qualitatively different from RLR signaling from mitochondria, with activation of IRF-1 and 3 and induction of a subset of ISGs [41]. Therefore, we were interested in identifying subcellular localization of the HIV RNA after transfection into PBMCs. To address this issue, we transfected cells with FAM-labelled HIV Tar and stained with mitotracker (mitochondria) and catalase (peroxisomes) to achieve organelle-specific staining. The cells were fixed and examined by confocal microscopy (Fig. 4). In agreement with previous publications, we found that synthetic oligos transfected into cells exhibited distinct punctate locations [27], [42]. This is in contrast to the more even distribution of the RNA observed after transfection of siRNAs, which are generally shorter [43]. We observed that the HIV-derived RNA oligo localized to distinct areas in the cell, and did not display any co-localization with mitochondria (Fig. 4A). By contrast, in a large percentage of the cells we found remarkable co-localization between HIV Tar RNA and the peroxisome marker catalase (Fig. 4B). This was seen at both 1 and 3 h post RNA transfection (Fig. 4 and data not shown). Thus, HIV-derived secondary structured RNA co-localizes with peroxisomes but not mitochondria after transfection into PBMCs.

### HIV RNA induces innate responses dependent on RIG-I and MAVS

**Figure 5:**
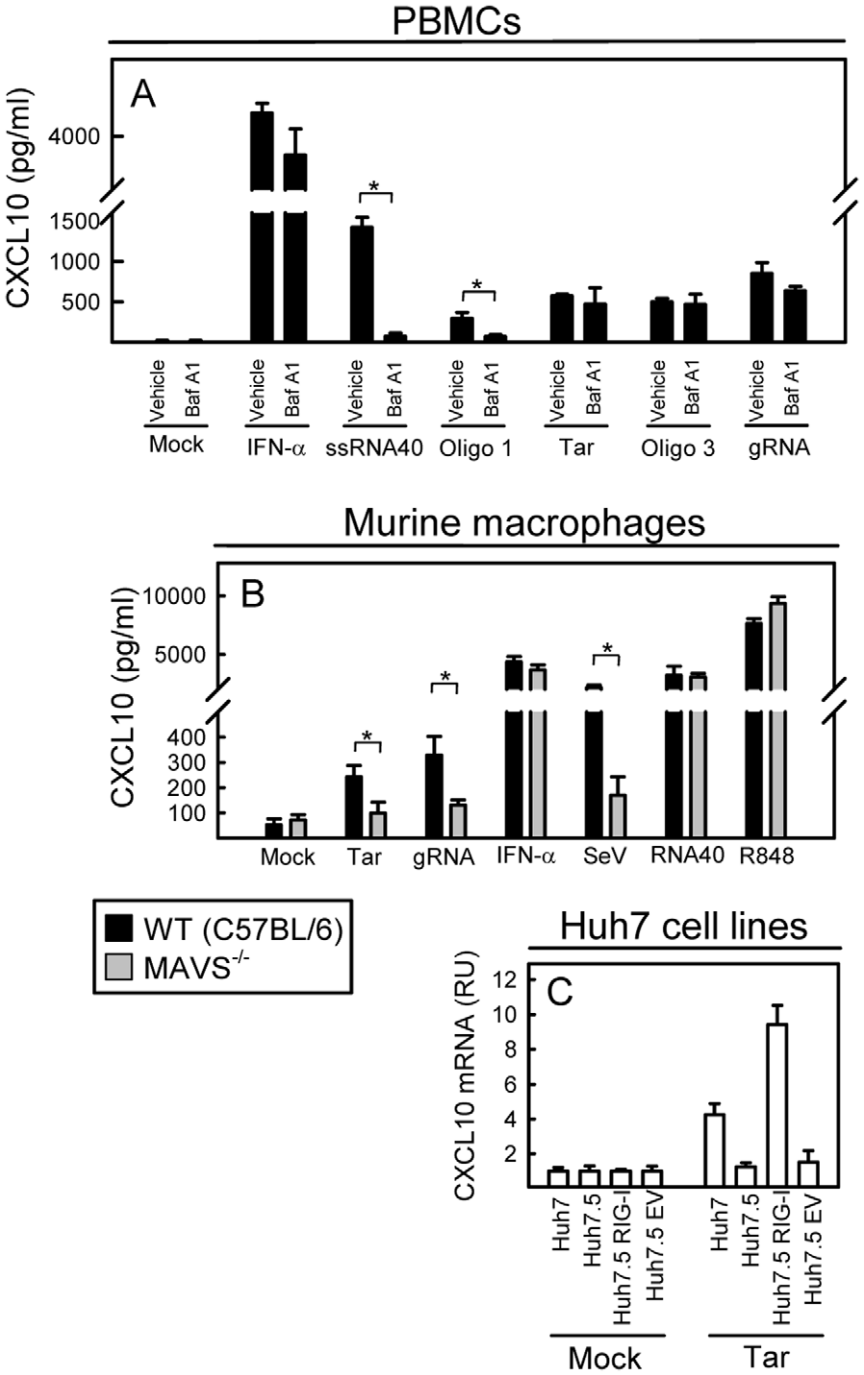
The innate immune response induced by HIV genomic RNA or RNA oligos is dependent on RIG-I and MAVS. (A) PBMCs were treated with bafilomycin A1 (0.5 µM) as indicated 15 min prior to stimulation with genomic HIV RNA, RNA oligos, or ssRNA40 (all 2 µg/ml). IFN-α was included as a positive control (10 ng/ml). Supernatants were harvested 18 h post stimulation for measurement of CXCL10. (B) BMMs from C57BL/6 wildtype and MAVS−/− mice were stimulated with genomic HIV RNA (2 µg/ml), Tar (2 µg/ml), Sendai virus (MOI 1), IFN-α (10 ng/ml), ssRNA40 (2 µg/ml), or R848 (2 µg/ml). Supernatants were harvested after 16 h and CXCL10 was measured by ELISA. UT, untreated cells. Data are shown as means of triplicates +/− st.dev. (C) Huh7, Huh7.5 (RIG-I mutant), Huh7.5 EV (empty vector), and Huh7.5 RIG-I cells were transfected with Tar RNA (2 µg/ml), or subjected to mock transfection with Lipofectamine 2000. Total RNA was harvested 6 h later and CXCL10 mRNA levels were analysed by qPCR. Data are shown as means of triplicates +/− st.dev. Similar results were obtained in two or three independent experiments. Mock, Lipofectamine 2000 alone. RU, relative units *, p<0.05.

To gain insight into the molecular mechanisms of HIV RNA sensing, we incubated PBMCs with bafilomycin A1 (which inhibits endosomal acidification and hence inhibits signaling by the endosomal TLRs – TLR3, 7, 8, and 9) prior to stimulation with HIV genomic RNA and HIV RNA oligos. Cell culture supernatants were harvested for measurement of CXCL10 (Fig. 5A). As expected, bafilomycin abrogated CXCL10 production in response to the TLR7/8 agonist ssRNA40. Likewise, we observed a strong inhibition of the response induced by the non-structured oligo 1. However, in the case of the HIV RNA oligos with higher order secondary RNA structure, bafilomycin only exerted a minor inhibitory effect on CXCL10 production. Importantly, genomic RNA displayed the same insensitivity to bafilomycin A1 as the secondary structured oligos by inducing CXCL10 in a manner not inhibited by bafilomycin A1. Thus, the HIV RNA tested induced innate immune responses dependent and independent of endosomal TLRs with the secondary structured RNA and genomic RNA stimulating CXCL10 expression largely independently of these PRRs.

Given the observed TLR-independency of the response triggered by genomic HIV RNA and the secondary structured oligos, we were interested in examining the possible contribution from alternative PRRs in sensing of HIV RNA and induction of an ISG response. Since we were particularly interested in the role of the RLR pathway, we focused on MAVS, an adaptor molecule essential for RLR signaling [39]. We generated mouse bone-marrow-derived macrophages (BMM)s from C57BL/6 wildtype and MAVS−/− mice and stimulated the cells with HIV Tar and viral genomic RNA. Importantly, both stimuli induced expression of CXCL10 in the wild-type BMMs, but this response was strongly inhibited in cells deficient in MAVS, and hence RLR signaling (Fig. 5B). Measurement of CXCL10 expression after stimulations with IFN-α Sendai virus, ssRNA40, and R848 showed that the MAVS-deficient cells did have the capacity to induce CXCL10 and were specifically defect in the response to a well-characterised RIG-I activating virus (Fig. 5B). In order to examine if RIG-I could be responsible for the observed MAVS dependent activation of innate immune responses, we used Huh7 cells and the derived cell line Huh7.5 with defect RIG-I function [44]. As seen in Fig. 5C, transfection with HIV Tar led to induction of CXCL10 in the parental Huh7 cells, which was not observed in the RIG-I mutant cell line Huh7.5. Reconstitution of the cells with RIG-I restored the response to HIV Tar RNA (Fig. 5D). In summary, these data demonstrate that secondary-structured HIV RNA is recognized by RIG-I, which induces innate immune response through MAVS.

## Discussion

Recently, innate immune responses have been appreciated to play an important role in both control and pathogenesis of HIV infection [13], [29], [36], [45]. However, knowledge on specific HIV PAMPs and cellular PRRs responsible for inducing innate immune responses has remained relatively sparse. Here we have identified the RIG-I/MAVS pathway as a sensor system of HIV genomic RNA in PBMCs. In contrast, only a minor contribution from endosomal TLRs was revealed. We investigated the importance of secondary structures in the HIV genome and found that the response to secondary structured but not unstructured RNA oligos derived from the HIV-1 genome mirrored the response to genomic RNA. Finally, we observed co-localization between HIV Tar and peroxisomes suggesting a possible involvement of this organelle as a signaling platform to NF-κB and IRFs in activation of innate immune responses directed against HIV.

Since most previous studies have been conducted mainly in cells that are not natural hosts for HIV-1 infection and have been based on short synthetic RNA oligos (often on the unnatural phosphorothioate backbone) derived from the HIV genome [16], [29], our study is unique in examining virion-derived HIV genomic RNA in PBMCs. Given that the HIV genome is highly structured [38], such secondary structures may influence, how HIV RNA is recognized in vivo by cellular PRRs. In the present study we found that genomic HIV RNA induced the ISG CXCL10 and also to a lesser extent IFN-β in human PBMCs. When we compared the early response to different HIV oligos with varying degrees of secondary structure, the more structured oligos induced higher responses and displayed a PRR requirement resembling that of genomic RNA. Specifically, we found that both genomic RNA and synthetic HIV Tar induced CXCL10 expression in a RIG-I/MAVS-dependent manner, whereas the non-structured RNA induced a pathway dependent on endosomal acidification, most likely TLR7. The recognition of HIV RNA by RIG-I is in agreement with a recent report published by Hiscott and associates [34]. These authors reported a stronger IFN-response to monomeric HIV RNA than to dimeric HIV RNA [34], but the degree of secondary structure and possible implications of such differences were not addressed. Given that full-length genomic HIV RNA, is endowed with secondary modifications, including capping and a poly-A-tail, which normally prevent mRNA from being recognized by the innate immune system, it remains unresolved how the HIV genome is recognized as foreign. One possibility is that genomic HIV RNA may be organized with a higher degree of secondary structures than most mRNA, the latter of which may be associated with host factors that reduce secondary structures and transport mRNA directly to the translational complex in the endoplasmic reticulum.

During recent years, growing evidence suggests that subcellular localization of signaling platforms and association with cellular organelles play important roles in the timing and shaping of inflammatory responses. For instance, mitochondria have been demonstrated to play an essential part in the response to both RNA and DNA through the involvement of the adaptor MAVS linked to mitochondria and essential in signaling complexes downstream of RLRs [25], [39]. This allows for integration of signals of viral infection or cellular stress, which may need coordination of both pro-inflammatory and pro-apoptotic responses. Likewise, the adaptor stimulator of interferon genes (also called STING) has been demonstrated to be associated with membranes of both the endoplasmic reticulum, the Golgi apparatus, and perinuclear vesicles [40], [46]. Therefore, we were interested in visualizing the cellular fate of the transfected HIV oligos, and in particular their possible co-localization with cellular organelles in the cytosol. By confocal microscopy and colorization of relevant cellular organelles, we observed that HIV Tar oligos tended to assemble in specific areas, which also stained for markers for peroxisomes but not mitochondria. This finding suggests that the Tar oligo used may interact with specific molecules, including adaptor proteins or receptors, linked to peroxisomal structures. Indeed, a recent report by Kagan and associates have implicated peroxisomes in immune defences by demonstrating localization of MAVS to peroxisomal membranes, where it is essential for inducing a rapid IFN-independent response, including ISGs, and dependent on the transcription factors IRF-1 and 3 [41]. The authors suggested that peroxisomal MAVS may induce a rapid antiviral defence programme serving to provide short term protection, until mitochondrial MAVS is able to induce an IFN-dependent signaling pathway with delayed kinetics aiming at amplifying and stabilizing the antiviral response [41]. In the present study, we found that HIV RNA co-localized with peroxisomes and activated IRF-1 and 3, as well as NF-κB and p38. By contrast, co-localization between RNA and mitochondria was not observed, even at later time points (data not shown). The involvement of signaling from the peroxisome platform in response to HIV RNA was further supported by the gene expression pattern dominated by ISGs with lower induction of type I and III IFN. Thus, during HIV infection, genomic RNA present in the cytosolic compartment soon after viral entry may induce a rapid antiviral response via peroxisomal MAVS, whereas other viral PAMPs present later during the replication cycle may induce a more sustained IFN-dependent response involving mitochondrial MAVS.

Previous studies using the synthetic HIV RNA oligo ssRNA40 have demonstrated MyD88-dependent signaling mediated by TLR7 in pDCs resulting in production of IFN-α [29]. Several other studies have also provided evidence for the involvement of additional endosomal TLRs, including TLR3 and TLR9 in response to retroviruses [47], [48]. However, the confinement to the endosomal compartment of these receptors requires either endocytic uptake of viral material, including HIV RNA, from the extracellular environment, or alternatively transport of these structures from the cytosol into the endolysosomal compartment by autophagy [49]. Using bafilomycin A1, an inhibitor of endolysosomal acidification and TLR signaling, we observed no significant inhibition of the innate response to genomic RNA or secondary structured RNA oligos. Together with the strong reduction of CXCL10 synthesis observed in cells deficient in RLR-triggered signaling through MAVS, this corroborates the finding that HIV genomic RNA is sensed primarily by cytosolic RIG-I rather than endocytic TLRs in PBMCs. This is in agreement with a study by Solis *et al*. in which genomic HIV RNA was reported to activate RIG-I and elicit a type I IFN response [34]. However, the authors went on to show that de novo HIV replication in monocyte-derived macrophages did not induce substantial amounts of IFN. This could be attributed at least in part to an HIV protease-dependent targeting of RIG-I to lysosomes resulting in inhibition of IRF-3 phosphorylation and decreased expression of IFN and ISGs [34]. Reduced levels of IRF3 have also been demonstrated in T cells in the presence of productive HIV replication. Interestingly this observation could be extrapolated to HIV-infected patients, in whom reduced IRF3 levels in CD4+ T cells were found only in acute HIV infection but not in long-term non-progressors [50]. Evidence for the involvement of both TLR- and cytosolic HIV-sensing depending on the cell type studied has been reported by Lepelley *et al*. In a study where HIV-infected cells were co-cultured with target cells (PBMCs or pDCs), it was demonstrated that virus-infected cells in contrast to cell-free virions elicited a strong IFN and ISG response [32]. The authors suggested that incoming viral material may be taken up by target cells via endocytosis, thereby allowing direct delivery of the viral RNA genome to endosomal TLRs. Indeed, recognition was reported to occur largely through TLR7 in pDCs and pDC-like cells rather than through cytosolic sensors [32], although evidence was also provided for the participation of cytosolic IRF3 activation in the absence of TLR7. However, the cytosolic HIV sensor involved was not definitively identified, but possible candidates are RIG-I or DNA receptors. Taken together, these studies together with our work illustrate that important cell type differences exist in sensing of HIV PAMPs and suggest that the PRR(s) involved may be dependent on the mechanism, by which the virus and viral replication products are presented to the cell, as well as the evasion strategies employed by the virus.

The establishment of RIG-I in recognition of the HIV RNA genome in the present work is novel and contributes, together with the report by Solis *et al.* reaching similar conclusions [34], to extending the current knowledge on innate immune sensing of HIV PAMPs by host PRRs. This finding may also have important implications for therapeutic aspects of HIV infection, both in vaccine design and in future development of novel antiretroviral treatment strategies. From a clinical perspective, it has recently been reported that chloroquine therapy during chronic HIV infection reduces immune activation, suggesting a role of endosomal TLR signaling induced by HIV [51]. In a similar manner, RIG-I may represent a novel target of antiretroviral treatment. Depending on whether RIG-I turns out to play beneficial roles for the host or the virus, targeting this PRR with either agonists or antagonists could have impact on host restriction of viral replication, or alternatively inhibit chronic immune activation, hence affecting the immunopathogenesis and preventing the progression to immunodeficiency during chronic HIV infection.

There is now evidence that HIV-1 has the potential to stimulate the innate immune system through its RNA, DNA, and capsid [16], [34], [36], [37]. However this virus seems to be capable of efficiently evading these sensing systems. It will be interesting to learn if RIG-I is also operative during actual HIV infection in natural host cells and to determine to what extent this PRR system contributes to activation of early defence mechanisms against HIV.

## Materials and Methods

### Ethics statement

The work does not contain experiments using living animals. Experiments involving cells from mice sacrificed prior to any experimental procedures do not require permission according to Danish law (The Animal Protection Law). The work does contain human studies. Approval was received from the local ethical committee (Committee for Research Ethics for Mid-Jutland County, permission number M-20110108) and informed written consent from all participating subjects was obtained.

### Cells

The primary human cell populations used were PBMCs, and monocyte-derived macrophages. PBMCs were isolated using Ficoll-Plaque purchased from GE Healthcare. Briefly, the blood was placed under a layer of Ficoll-plaque and centrifuged at 600× g for 30 min. Cells from the interphase layer was harvested, washed twice in phosphate-buffered saline, and resuspended in RPMI-1640 medium containing 10% heat-inactivated FCS, and antibiotics (penicillin and streptomycin). For experiments, cells were seeded in 96-, 24- or 6-well plates at a density of 2×10^5^, 1×10^6^, or 4×10^6^ cells per well, respectively, and left for at least 6 hours before further treatment.

The human hepatocarcinoma cell line Huh7, and the derived cell lines Huh7.5, Huh7.5 EV (empty vector) and Huh7.5-RIG-I (kindly donated by Ralf Bartenschlager, Heidelberg, Germany) were maintained in DMEM supplemented with 10% FCS. For the latter 2 cell lines, the medium was supplemented with 250 µg/ml of G418 (Roche). For experiments, 7×10^5^ cells were seeded per well in 6-well plates and left 3–4 h prior to further treatment.

For generation of monocyte-derived macrophages, PBMCs were isolated from blood (leukocyte-rich buffy coats). Mononuclear cells were allowed to adhere onto plastic six-well plates (Falcon Multiwell; BD Biosciences) or plastic 24-well plates for 1 h at 37°C in RPMI 1640 medium supplemented with penicillin (0.6 µg/ml), streptomycin (60 µg/ml), glutamine (2 mM) and HEPES (20 mM). After monocyte binding, non-adherent cells were removed and the wells were washed twice with PBS, pH 7.4. Adherent cells were then grown for 7–8 days in Macrophage-SFM medium (Life Technologies) supplemented with antibiotics and granulocyte–macrophage colony-stimulating factor (GM-CSF; Nordic Biosite) at 10 ng/ml. GM-CSF-containing medium was removed from the cells 1 day before further stimulation [52]. The isolated cells were determined to be macrophages by their typical morphology and cell-surface CD14 expression [52]. Cells from individual blood donors were grown separately, but after stimulation experiments, they were pooled.

Mouse BMMs were obtained as follows: femur and tibia were surgically removed from C57BL/6 wildtype and MAVS-deficient mice (kindly provided by Professor Z.J. Chen, Southwestern Medical Center, Dallas Texas), freed of muscles and tendons, and briefly suspended in 70% ethanol. Ends were cut, the marrow was flushed with 10% RPMI 1640, and the cell suspension was filtered over a 70-µm cell strainer (BD Falcon) and centrifuged for 5 min at 1330 rpm. After 2 washes, cells were resuspended at 2×10^5^ cells/ml in RPMI 1640 with 10% FCS and GM-CSF (10 ng/ml), seeded in bacteriological petri dishes, and incubated at 37°C with 5% CO_2_ and media changed after 3 and 5 days. On day 7, adherent cells were harvested from the dishes with medium containing GM-CSF (10 ng/ml). The cells were centrifuged, washed, and resuspended in RPMI 1640, 10% FCS, and GM-CSF (20 ng/ml), and examined by flow cytometry for expression of CD11b and CD11c (data not shown). For *in vitro* experiments, the cells were used at a concentration of 1.0×10^6^ cells per well in 96-well plates in 100 µl medium.

### HIV genomic RNA and RNA oligos

HIV genomic RNA was isolated as previously described [38], [53]. Briefly, the virus was treated with DNase I and RNase Cocktain (RNase T1 and RNase A, Ambion) to remove extra-virion nucleic acids, and then subjected to subtilisin digestion. The lysates were phenol∶chloroform∶isoamyl alcohol-extracted and ethanol-preciptated. The RNA was re-suspended in water and used for experiments. The synthetic RNA oligos used were ssRNA40 (InvivoGen), 5′-GCCCGUCUGU UGUGUGACUC-3′; Oligo 1, 5′-AGGAAGAAGC GGAGACAGCG ACGAAGAGCU CAUCAGAACA GUCAGACUCA -3′; Oligo 2/Tar, 5′-UCUCUGGUUA GACCAGAUCU GAGCCUGGGA GCUCUCUGGC UAACUAGGGA-3′; Oligo 3, 5′- GUCAACAUAA UUGGAAGAAA UAUGUUGACU CAGAUUGGUU GCACUUUAAA UUUUCCAAUU -3′; Oligo 4, 5′-GAGTAGTAGA ATCTATGAAT AAAGAATTAA AGAAAATTAT AGGACAGGTA–3′; Oligo 5, 5′-CACGGACAAT GCTAAAACCA TAATAGTACA GCTGAACACA TCTGTAGAAA-3′ (all DNA Technology) (Fig. 2A). ssRNA40 is on phosphorothioate backbone, whereas oligos 1–5 were synthesized with 3′ and 5′ OH-ends and on phosphodiester backbone. Secondary structures of the RNA oligos used were predicted using the RNAfold Webserver (rna.tbi.univie.ac.at/cgi-bin/RNAfold.cgi).

### RNA transfection

RNA oligos or genomic HIV RNA was transfected into cells using Lipofectamine 2000 (Invitrogen). RNA and Lipofectamine 2000 complexes were prepared in OptiMEM in the ratio of 10 µl lipofectamine per 4 µg RNA. After 20 min of incubation, the RNA mixture was added to cells resulting in a final concentration of RNA between 1 and 3 µg/ml.

### Reagents for cell stimulation, infections and transfections

Control stimuli were added directly to the media and included R848 (TLR7/8 agonist, 5 µg/ml), ssRNA40 (TLR7 agonist, 2 µg/ml), ssRNA41 (negative TLR7 control, 2 µg/ml) (all from InvivoGen), IFN-α (10 ng/ml, PBL InterferonSource), IFN-γ (100 ng/ml, R&D Systems), Sendai virus (MOI 1). Inhibition of endosomal acidification, and thereby TLR3, 7/8, and 9 signaling was achieved by pre-incubating the cells with bafilomycin A1 (0.5 µM, Sigma Aldrich) 15 min prior to stimulation. Sendai virus was used at multiplicity of infection (MOI) of 1.

### RNA isolation and RT

Macrophages were washed once with PBS and lysed, and total cellular RNA was recovered using an RNA purification kit (Macherey-Nagel NucleoSpin RNA II or Qiagen Midi kit) as described by the manufacturer. Total RNA from PBMCs was recovered by Trizol (Invitrogen) as described by the manufacturer. Purified RNA was dissolved in water and stored at −80°C until analysis. cDNA synthesis was performed with 1 to 2 µg RNA by using a Moloney murine leukemia virus reverse transcriptase kit (Invitrogen) according to the manufacturer's instructions with an oligo(dT)_18_ primer (DNA Technology, Aarhus, Denmark) [54] or as described previously [55].

### Quantitative real-time PCR

cDNA obtained from macrophages was quantified by TaqMan real-time PCR using primers and probes from Applied Biosystems for IFN-β, IFN-λ1, CXCL10, RIG-I, TNF-α, and IL-1β, as previously described [55]. cDNA from PBMCs was amplified by realtime PCR using primers for GAPDH, IFN-β, and CXCL10 (DNA Technology), as previously described [54].

### Detection of Phosphoproteins

For detection of the phosphorylation status of IκBα and p38 Luminex technology was used. Briefly, the filter plate was washed with assay buffer, and 50 µl of freshly vortexed antibody-conjugated beads were added to each well. The plate was washed with assay buffer, and samples of whole-cell lysates were added. After a brief shake (30 s at 1100 rpm), the plate was incubated at 4°C overnight in the dark with light shaking (300 rpm). After one wash step, 25 µl of the detection antibody was added to each well, and the plate was shaken and incubated as above. Subsequently, the plate was washed and incubated for 30 min with 50 µl of a streptavidin-PE solution with shaking (30 s at 1100 rpm, 10 min at 300 rpm). Finally, the plate was washed, 125 µl of assay buffer was added to each well, and the plate was shaken for 10 s at 1100 rpm and read immediately on a Bio-Plex reader.

### IRF DNA-binding activity

ELISA-based measurement of DNA-binding activity of nuclear IRF family members was performed using TransAM, following the protocol of the manufacturer (Active Motif). Briefly, 5 µg of nuclear extract, isolated with the Nuclear Extract Kit (Active Motif), was used per sample in duplets in a 96-well plate pre-coated with consensus oligonucleotides for the IFN-stimulated response element. After washing to remove nonspecific binding, antibodies specific for IRF-1, -3, and -7 were added. After antibody binding, the plate was washed again before adding a HRP-conjugated secondary antibody. The peroxidase substrate was added, and colorimetric change was measured at an optical density of 450 (OD450).

### Measurement of cytokines

Human and murine CXCL10 were detected by Cytoset (Invitrogen) and Duoset (R&D Sytems), respectively. IFN-β was measured by an ELISA kit from PBL InterferonSource. Analysis was performed as described by the manufacturers. The result was visualized by the tetramethylbenzidine system (R&D Systems or KemEnTec) and quantified by reading at 450 nm and 650 as reference. Human IFN-α, IL-6, and TNF-α were measured by Luminex technology, using a custom-made three-plex kit, purchased from Bio-Rad (Hercules, CA), following the instructions of the manufacturer. Detection limits for the cytokine analysis assays: CXCL10, 17 pg/ml; IFN-β, 2 U/ml; IFN-α, 25 pg/ml; TNF-α, 13 pg/ml; IL-6, 20 pg/ml.

### Confocal microscopy

Human PBMCs were plated at 1×10^6^ cells/ml overnight in RPMI containing 10% FCS onto 12 mm coverslips. Cells were transfected, with 1.2 µg/ml FAM-labelled HIV-Tar RNA at a RNA∶Lipofectamine 2000 (Invitrogen) ratio of 1∶2.5 (mass∶volume) for 180 min. Cells were subsequently fixed with methanol at −20°C for 5 min and stained with anti-Catalase (Abcam) to label peroxisomes or with DAPI to label nuclear DNA. To label mitochondria 200 nM Mitotracker Far Red (Invitrogen) was added to cells 15 min prior to fixation. Images were gathered using Zeiss LSM 710 confocal microscope, 63×1.4 oil objective. Image processing was performed by MacBiophotonics Image J.

### Statistical analysis

The data are presented as means ± standard deviations (st.dev.). The statistical significance of differences between observations was estimated with the Wilcoxon rank sum test. *P* values of <0.05 were considered statistically significant.
